# Predictors, Moderators, and Mediators Associated With Treatment Outcome in Randomized Clinical Trials Among Adolescents With Depression: A Scoping Review

**DOI:** 10.1001/jamanetworkopen.2021.46331

**Published:** 2022-02-01

**Authors:** Darren B. Courtney, Priya Watson, Karolin R. Krause, Benjamin W. C. Chan, Kathryn Bennett, Meredith Gunlicks-Stoessel, Terri Rodak, Kirsten Neprily, Tabitha Zentner, Peter Szatmari

**Affiliations:** 1 Centre for Addiction and Mental Health, Toronto, Ontario, Canada; 2 Department of Psychiatry, University of Toronto, Toronto, Ontario, Canada; 3 Family Physician, Toronto, Ontario, Canada; 4 Department of Health Research Methods, Evidence and Impact (formerly Clinical Epidemiology and Biostatistics), McMaster University Faculty of Health Sciences, Hamilton, Ontario, Canada; 5 Department of Psychiatry and Behavioral Sciences, University of Minnesota, Minneapolis; 6 Centre for Addiction and Mental Health Education, Centre for Addiction and Mental Health, Toronto, Ontario, Canada; 7 School and Applied Child Psychology, University of Calgary, Calgary, Alberta, Canada; 8 Margaret and Wallace McCain Centre for Child, Youth and Family Mental Health, Centre for Addiction and Mental Health, Toronto, Ontario, Canada

## Abstract

**Importance:**

The application of precision medicine principles for the treatment of depressive disorders in adolescents requires an examination of the variables associated with depression outcomes in randomized clinical trials (RCTs).

**Objective:**

To describe predictors, moderators, and mediators associated with outcomes in RCTs for the treatment of depressive disorders in adolescents.

**Evidence Review:**

A scoping review of RCTs for the treatment of depression in adolescents was conducted. Databases searched included MEDLINE, Embase, APA PsycInfo, and CINAHL. Included publications tested predictors, moderators, and/or mediators associated with depression symptom outcomes (eg, symptom reduction, response, remission) in RCTs pertaining to the treatment of adolescents, ages 13 to 17 years. Predictors were defined as variables that were associated with depression outcomes, independent of treatment group. Moderators were defined as baseline variables that were associated with differential outcomes between treatment groups. Mediators were defined by a formal mediation analysis. In duplicate, variables were extracted and coded with respect to analysis type (univariable or multivariable), statistical significance, direction of effect size, reporting of a priori hypotheses, and adjustment for multiple comparisons. Aggregated results were summarized by variable domain and RCT sample.

**Findings:**

Eighty-one articles reporting on variables associated with outcomes across 33 RCTs were identified, including studies of biological (10 RCTs), psychosocial (18 RCTs), and combined (4 RCTs) treatments as well as a service delivery model (1 RCT). Fifty-three variable domains were tested as baseline predictors of depression outcome, 41 as moderators, 19 as postbaseline predictors, and 5 as mediators. Variable domains that were reported as significant in at least 3 RCTs included age, sex/gender, baseline depression severity, early response to treatment, sleep changes, parent-child conflict, overall psychopathology, suicidal ideation, hopelessness, functional impairment, attendance at therapy sessions, and history of trauma. Two publications reported a priori hypotheses and adjustment for multiple comparisons, both finding that baseline depression severity and family conflict were associated with poorer outcomes.

**Conclusions and Relevance:**

This review identified commonly researched variables requiring more scrutiny as well as underresearched variables to inform future study designs. Further efforts to discover predictors, moderators, and mediators associated with treatment response have great potential to optimize care for adolescents with depression.

## Key Points

**Question:**

What do randomized clinical trials for treatment of adolescents with depressive disorders report about the predictors, moderators, and mediators associated with outcomes?

**Findings:**

In this scoping review of 33 RCTs with results described across 81 publications, variable domains reported as significant in at least 3 RCTs with respect to depression outcomes included age, sex/gender, baseline depression severity, early response to treatment, sleep changes, parent-child conflict, overall psychopathology, suicidal ideation, hopelessness, functional impairment, attendance at psychotherapy sessions, and history of trauma. A small minority of publications reported efforts to minimize bias through a priori hypothesis testing and adjustment for multiple comparisons.

**Meaning:**

Variables identified in this review can be incorporated into rigorous research designs to further test the optimization of care for adolescents with depression.

## Teaser

This scoping review describing findings of predictors, moderators, and mediators associated with outcomes in randomized clinical trials for the treatment of depressive disorders in adolescents.

## Introduction

Depressive disorders in adolescents (DD-A) are prevalent,^1^ impairing,^2,3^ and associated with suicide.^4,5^ In the United States, rates of depressive symptoms and suicide in adolescents have increased over the past 10 to 15 years.^6,7^ Current treatment approaches have limited benefit.^8,9^ The application of precision medicine hopes to improve outcomes by offering “treatment strategies that take individual variability into account.”^10^ Clinicians treating DD-A are expected to practice precision medicine.^11^ Researchers of DD-A treatment also need to understand how clinical factors are associated with outcomes to guide the development and testing of new treatment approaches.^12^ Information from randomized clinical trials (RCTs) can elucidate these factors, as rigorous data collection is conducted at set time points under controlled treatment conditions. A good understanding of the variables associated with depression severity outcome in RCTs for the treatment of DD-A can indicate what works for whom and how.^12,13^ Two previous evidence syntheses that have examined such variables^14,15^ have included a very limited set of relevant studies.

An up-to-date, systematic, and comprehensive examination of what is currently known about predictors, moderators, and mediators derived from RCTs for the treatment of DD-A can inform the extent to which clinicians can practice precision medicine and guide trialists on interventions targeting specific mechanisms of action. The aims of this scoping review were to (1) identify the predictors, moderators, and mediators that have been studied to date in published RCTs of treatment for DD-A, (2) map out the reported findings from their analyses to guide further hypothesis testing, and (3) describe the extent to which a priori hypotheses and adjustments for multiple comparisons are reported in published analyses.

## Methods

Methods are detailed in our registration,^16^ preprint,^17^ and published protocol.^18,19^ A scoping review design was chosen for this study’s aims, as it is most appropriate for mapping out broad concepts, describing the extent of the available literature, and identifying gaps.^20,21^ This is in contrast to a systematic review design, intended to summarize the literature to answer specific questions, or meta-analyses, in which data are reanalyzed.^21^ We applied scoping review methods outlined by the Joanna Briggs Institute^21^ and followed the Preferred Reporting Items for Systematic Reviews and Meta-analyses Extension for Scoping Reviews (PRISMA-ScR) checklist for scoping reviews.^20^

Inclusion criteria were English-language RCTs that assessed treatments of depressive disorders in adolescents (ages 13-17 years) in which (1) depression was defined as diagnoses of major depressive disorder, dysthymia/persistent depressive disorder, or depressive symptoms more severe than an established cutoff on a validated measure of depression symptom severity; (2) treatment interventions included biological interventions (eg, antidepressants), psychosocial interventions (eg, psychotherapy), or service delivery models (eg, collaborative care arrangements between mental health specialists and primary care); (3) a test of any predictor, moderator, or mediator associated with depression outcomes was conducted. Exclusion criteria were RCTs evaluating bipolar depression, peripartum depression, premenstrual dysphoria, minor depression, or seasonal affective disorder; RCTs targeting the prevention of depression or recurrence of depression; and economic analyses. Additional exclusion criteria, which were protocol deviations, were conference abstracts, dissertations, and studies with sample sizes less than 50.

We used a planned search strategy (eAppendix in Supplement 1) with the following databases: MEDLINE, Embase, APA PsycInfo, and CINAHL. The search date limits were from inception of the respective database to February 6, 2020. Source selection was performed by 3 investigators (D.B.C., P.W., and B.W.C.C.) with established interrater reliability (Fleiss κ = 0.93). Results were extracted and coded in duplicate by 3 of us (D.B.C., P.W., and K.R.K.) with respect to reported analysis type (univariable or multivariable), statistical significance, direction of effect size, reporting of a priori hypotheses, and adjustment for multiple comparisons.

Definitions of predictors, moderators, and mediators from the literature were applied to categorize variables and analyses. Baseline predictors were defined as baseline variables that were associated with depression outcomes, independent of treatment group.^13^ Moderators (ie, effect modifiers) were defined as baseline variables that were associated with differential outcomes between treatment groups.^13,22,23^ Postbaseline predictor variables (including time-varying covariates) were defined as variables measured during or after treatment that were associated with depression outcomes, independent of treatment groups.^13^ Mediators were defined by an analysis of (1) the relationship between an independent variable correlated with treatment group and a postbaseline mediating variable, (2) the relationship between the mediating variable and a dependent outcome, and (3) the extent to which these 2 relationships account for the direct relationship between the independent variable and dependent variable.^13,23^

For each paper, 2 of 3 investigators (D.B.C., P.W., and K.R.K.) independently extracted the findings in duplicate with respect to end point depression outcomes, ie, outcomes relating to the measurement of depressive symptoms using an evaluator-rated scale (eg, the Childhood Depression Rating Scale–Revised [CDRS-R]^24^) or self-rated scale (eg, Mood and Feelings Questionnaire^25^). The most common examples of depression outcomes were continuous outcomes of symptom reduction on a scale score over time or dichotomous outcomes of response or remission.^19^ Regardless of treatment exposure, response was most often defined as a specified percentage decrease in depression scale scores (eg, 50% reduction on the CDRS-R) or a rating of much improved or very much improved on the Clinician’s Global Impression–Improvement subscale.^26^ Remission was most often defined by an end point scale score below a specific cutoff or no longer meeting criteria for major depressive disorder.^19^ Greater reduction in symptom scale scores over time or greater proportions of responders and/or remitters represented favorable outcomes. Predictors, moderators, and mediators associated with other outcomes (eg, suicidal ideation, function) were not extracted in this review.

We created detailed tables describing the findings by publication, RCT sample size, and independent variable of interest. Given that *P* values were universally described across analyses and articles to characterize results, we categorized reported findings as significant or not significant, depending on the threshold set by the articles’ authors; most often this threshold was *P* ≤ .05, unless adjustments for multiple comparisons were made. Aggregated summary tables were created for the reporting of high-level findings from (1) secondary analyses relating to the same RCT sample and (2) predictor, moderator, and mediator variable thematic domains.^27^ We did not carry out our initial plan to extract effect sizes, which is more appropriate for focused individual patient data meta-analyses. In duplicate, we performed a preliminary risk of bias assessment relevant to secondary analyses; namely, with respect to (1) a priori model development and (2) correction for multiple testing.

## Results

Of 98 RCTs identified in total in our search, 33 RCTs (Table 1; eTable 1 in Supplement 2)^28,29,30,31,32,33,34,35,36,37,38,39,40,41,42,43,44,45,46,47,48,49,50,51,52,53,54,55,56,57,58,59,60^ reported results of at least 1 predictor, moderator, or mediator tested for association with depression outcome. Most interventions studied were antidepressants, psychotherapy, and their combination. Analysis of predictors, moderators, and mediators associated with outcomes were reported across the 81 individual publications associated with these RCTs (eTable 2 in Supplement 2),^28,29,30,31,32,33,34,35,36,37,38,39,40,41,42,43,44,45,46,47,48,49,50,51,52,53,54,61,62,63,64,65,66,67,68,69,70,71,72,73,74,75,76,77,78,79,80,81,82,83,84,85,86,87,88,89,90,91,92,93,94,95,96,97,98,99,100,101,102,103,104,105,106,107,108,109,110,111,112,113,114^ identified through the citation selection process shown in the Figure.

**Table 1.** Study Characteristics of Included Original Randomized Clinical Trials for the Treatment of Depressive Disorders in Adolescents

Variable	Studies, No. (%) (N = 33)
Region where first author is based	
North America	20 (61)
Europe	9 (27)
South America	2 (6)
Africa	1 (3)
Oceania	1 (3)
Sex distribution of participants	
More female than male participants	25 (76)
More male than female participants	1 (3)
Not reported	7 (21)
Sample size	
51-100	11 (33)
101-200	13 (39)
201-400	7 (21)
>400	2 (6)
Funding	
Nonindustry	25 (76)
Industry	7 (21)
Not reported	1 (3)
Recruitment setting	
Outpatient only	8 (24)
Community only	4 (12)
School only	3 (9)
Inpatient only	2 (6)
Primary care only	1 (3)
Combination	11 (33)
Not reported	4 (12)
Experimental intervention type	
Biological	
Antidepressant medications	8 (24)
Light therapy	2 (6)
Psychotherapy	
Cognitive behavioral therapy	8 (24)
Interpersonal therapy	3 (9)
Family therapy	2 (6)
Psychoeducation	1 (3)
Comparisons between therapies	4 (12)
Combination antidepressant with therapy	4 (12)
Service delivery model (eg, collaborative care)	1 (3)
Duration of randomized component of trial	
3 d to <8 wk	4 (12)
8 to 12 wk	23 (70)
>12 to 16 wk	5 (15)
>16 to 104 wk	1 (3)
Method of identifying depression as inclusion criteria	
*DSM* or *ICD* criteria	7 (21)
Cutoff on a scale score	6 (18)
Both	20 (61)

Abbreviations: *DSM*, *Diagnostic and Statistical Manual of Mental Disorders*; *ICD*, *International Classification of Diseases*.

**Figure.**
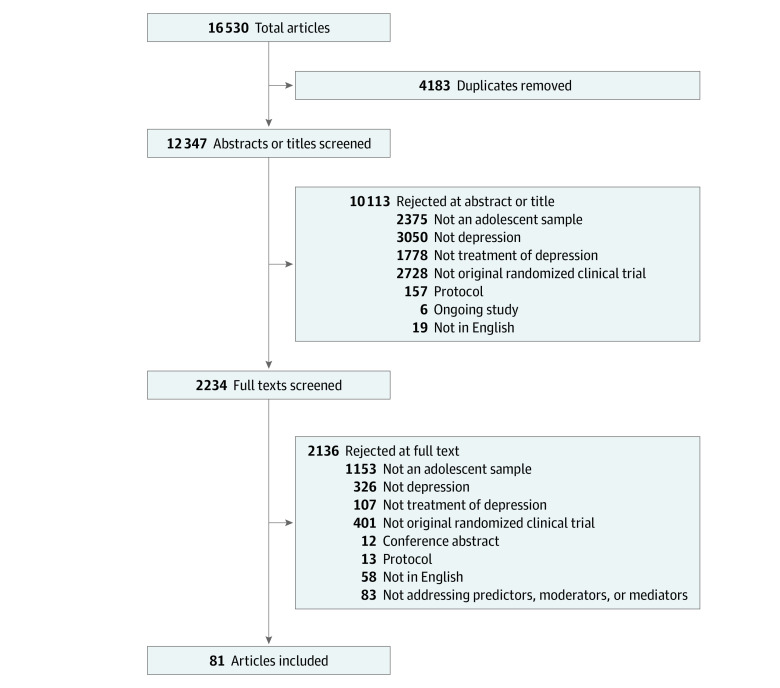
Study Flow Diagram

Across these 81 publications, a wide range of variables were tested as predictors, moderators, and mediators associated with depression outcome. Fifty-one publications (63%)^29,31,33,34,35,39,41,43,45,46,47,49,50,53,54,65,66,68,71,73,74,75,76,77,78,79,80,81,82,83,84,85,86,87,88,95,96,98,100,101,102,103,105,107,108,109,110,111,113,114,115^ tested baseline predictors, 45 (56%)^28,30,31,33,34,36,37,39,40,41,44,49,50,51,53,61,64,66,67,68,69,70,74,75,77,79,80,84,85,89,93,94,96,98,100,101,102,103,105,109,110,111,113,114^ tested moderators, 32 (40%)^32,33,37,38,42,45,47,51,52,53,65,69,71,78,80,81,87,88,91,92,95,96,97,99,100,104,105,106,107,108,109,112^ tested postbaseline predictors, and 5 (6%)^28,61,62,63,94^ conducted formal mediation analyses. Detailed tables listing relevant RCTs, individual publications associated with these RCTs, variables tested as predictors, moderators, and mediators, and analysis results are available in the supplementary materials (eTables 3-9 in Supplement 2).

Demographic variables tested included sex/gender; age; race and ethnicity; socioeconomic status (SES); rurality; body mass index/weight; lesbian, gay, bisexual, transgender, queer, and other sexual orientation or gender identity status; parental education; and single-parent household. No study differentiated definitions of sex (ie, sex assigned at birth) and gender (ie, current gender identity), nor did any study differentiate definitions of race relative to ethnicity.

Clinical symptom profile variables tested included factors related to young people’s depression profile and history, including baseline symptoms severity (with higher continuous baseline scale scores on a measure of depressive symptoms indicating higher severity), duration of current depressive episode at baseline, age of onset, number of previous depressive episodes, symptom profile (anhedonia, hopelessness, insomnia, melancholic symptoms, subsyndromal manic symptoms). They further included the presence of specific comorbidities, measures of overall psychopathology (including number of comorbid disorders), self-injurious thoughts and behaviors, and medication history.

Variables related to psychosocial context included general functioning (eg, completing tasks at school and home, getting along with others and engaging in recreational activities^116^), family functioning (eg, family’s ability to resolve conflict, support each other in regulating emotions, and solve problems^117^), psychological factors (eg, perfectionism, rumination, self-esteem, cognitive distortions, motivation), history of traumatic events (eg, physical or sexual abuse), recent stressful events (eg, death of loved one or moving), baseline coping skills (eg, avoidance strategies, problem-solving skills), and problem-solving orientation (eg, optimistic vs pessimistic perceptions about problem solving).

Treatment-specific factors tested as postbaseline predictors associated with depression outcome included attendance at psychotherapy sessions, cognitive behavioral therapy (CBT) homework completion, exposure to specific therapy components (eg, motivational interviewing, problem-solving), medication factors (eg, adherence, dosage), posttreatment symptoms, and early response (ie, a dichotomous outcome of response at a relatively early point in the study, such as 2-4 weeks post baseline). Variables tested as potential mediators included general functioning (as described previously), relationship functioning (ie, ability to get along with peers and family members), psychological factors (ie, perfectionism, motivation, dysfunctional attitudes, automatic thoughts), skill use (ie, relaxation skills, behavioral activation skills, social skills, and problem-solving skills), alliance with the therapist, and group cohesion for a group-based intervention.

### Baseline Predictors

Among 23 RCTs, 166 different variables were tested as baseline predictors, which we grouped into 53 domains. Baseline variables were analyzed as predictors associated with outcomes in 358 instances and, of these, 269 (75%) were reported as not significant. Baseline variable domains reported as significant with respect to depression outcome in at least 3 RCTs included age, depression severity, parent-child conflict, overall psychopathology, suicidal ideation, hopelessness, and functional impairment (Table 2).

**Table 2.** Baseline Variables Tested as Predictors of Depression Symptom Severity Outcomes in RCTs of Treatment for DD-A

Baseline variable	RCTs testing variable, No.	RCT analyses by result category, No. (%)				RCTs by direction of significant multivariable analyses, No.		
		Not significant	Significant on univariable analysis, not significant on multivariable analysis	Significant on univariable analysis, not challenged in multivariable analysis	Significant on any multivariable analysis	Greater depression symptom severity at end point	Less depression symptom severity at end point	Unclear
Demographic characteristics								
Female sex/gender	12	11 (92)	0	0	1 (8)	0	1	0
Older age	9	5 (55)	1 (11)	0	3 (33)	1	2	0
Race and ethnicity	6	6 (100)	0	0	0	0	0	0
Socioeconomic status	5	5 (100)	0	0	0	0	0	0
Single parent household	2	1 (50)	0	1 (50)	0	0	0	0
Rurality	1	1 (100)	0	0	0	0	0	0
Body mass index/weight	2	2 (100)	0	0	0	0	0	0
LGBTQ+ status	1	1 (100)	0	0	0	0	0	0
Parental education	1	1 (100)	0	0	0	0	0	0
Clinical profile								
Depression symptom severity	13	2 (15)	1 (8)	1 (8)	9 (69)	4	1	4
Anxiety symptoms	10	3 (30)	5 (50)	0	2 (20)	2	0	0
Overall psychopathology	7	3 (43)	0	0	4 (57)	4	0	0
Suicidal ideation	6	0	1 (17)	1 (17)	4 (66)	4	0	0
Hopelessness	5	0	2 (40)	0	3 (60)	3	0	0
Duration of depression	4	2 (50)	0	0	2 (50)	2	0	0
ADHD	4	3 (75)	1 (25)	0	0	0	0	0
Comorbid disruptive behavior	4	3 (75)	0	1 (25)	0	0	0	0
Age of onset of depression	3	2 (66)	1 (33)	0	0	0	0	0
Comorbid dysthymia at baseline	3	3 (100)	0	0	0	0	0	0
Lifetime history of suicide attempts	3	3 (100)	0	0	0	0	0	0
Nonsuicidal self-injury	3	1 (33)	0	0	2 (66)	2	0	0
No. of previous episodes	2	2 (100)	0	0	0	0	0	0
Low mood	2	1 (50)	1 (50)	0	0	0	0	0
Anhedonia	2	1 (50)	0	0	0	1	0	0
Obsessive-compulsive symptoms	3	2 (66)	0	0	1 (33)	1	0	0
Substance use	2	1 (50)	0	0	1 (50)	1	0	0
Medication history	2	1 (50)	0	0	1 (50)	0	1	0
Melancholic features	1	0	0	0	1 (100)	1	0	0
Observed symptoms	1	0	0	1 (100)	0	0	0	0
Depressive symptom clusters	1	0	1 (100)	0	0	0	0	0
Guilt	1	0	1 (100)	0	0	0	0	0
Somatic symptoms	1	0	1 (100)	0	0	0	0	0
Sleep disturbance	1	1 (100)	0	0	0	0	0	0
Appetite disturbance	1	1 (100)	0	0	0	0	0	0
Energy disturbance	1	1 (100)	0	0	0	0	0	0
Impairment in concentration	1	1 (100)	0	0	0	0	0	0
Psychomotor symptoms	1	1 (100)	0	0	0	0	0	0
Eating disorder	1	1 (100)	0	0	0	0	0	0
Manic symptoms	1	0	0	0	1 (100)	1	0	0
Psychotic symptoms	1	1 (100)	0	0	0	0	0	0
Psychosocial context								
General functional impairment	7	2 (29)	1 (14)	1 (14)	3 (43)	3	0	0
Poor family functioning	6	0	1 (17)	0	5 (83)	4	1	0
Trauma and/or childhood adversity	5	3 (60)	0	0	2 (40)	1	0	1
Psychological factors	5	1 (20)	1 (20)	1 (20)	2 (40)	1	0	1
Caregiver psychopathology	3	2 (66)	0	1 (33)	0	0	0	0
Treatment expectations	2	1 (50)	0	0	1 (50)	0	0	1
Coping or problem-solving approach	2	0	1 (50)	0	1 (50)	0	0	1
Verbal intelligence	1	1 (100)	0	0	0	0	0	0
Latitude of intervention site (proxy for seasonal affective disorder)	1	1 (100)	0	0	0	0	0	0
No. of adverse factors associated with outcomes	1	0	0	1 (100)	0	0	0	0
Pretreatment knowledge about depression	1	0	0	1 (100)	0	0	0	0
Recent stressful events	1	0	0	0	1 (100)	1	0	0
Family history of depression	1	1 (100)	0	0	0	0	0	0

Abbreviations: ADHD, attention-deficit/hyperactivity disorder; DD-A, depressive disorders in adolescents; LGBTQ+, lesbian, gay, bisexual, transgender, queer, and other sexual orientations or gender identities; RCT, randomized clinical trial.

### Moderators

Across 21 RCTs, 117 unique variables were tested with respect to moderators that we grouped into 41 domains. In these RCTs, baseline variables were tested as moderators in 197 instances; of these, 159 (81%) were reported as not significant. Baseline variable domains reported as significant when tested as moderators in at least 3 RCTs included sex/gender, depression severity, and history of trauma (Table 3).

**Table 3.** Baseline Variables Tested as Moderators of Depression Symptom Severity Outcomes in RCTs of Treatment of Depressive Disorder in Adolescents

Baseline variable	RCTs testing variable, No.	RCT analyses by result category, No. (%)				Nature of association, if significant on multivariable analysis
		Not significant	Significant on univariable analyses, dropped on multivariable analysis	Significant on univariable analysis, not challenged by multivariable analysis	Significant in multivariable analysis	
Demographic characteristics						
Female sex/gender	13	9 (69)	0	0	4 (31)	Females/girls more likely than males/boys to benefit from IPT-G relative to WL (Bolton et al,^33^ 2007), Reiki relative to WL (Charkhandeh et al,^36^ 2016), and duloxetine relative to placebo (Emslie et al,^40^ 2014); complex interaction between gender and marital discord in TADS (Amaya et al,^74^ 2011)
Older age	11	7 (64)	2 (18)	2 (18)	0	NA
White race	6	4 (66)	2 (34)	0	0	NA
Higher SES	4	3 (75)	0	0	1 (25)	High SES more likely to benefit from combination of fluoxetine and CBT or CBT alone relative to fluoxetine alone or placebo; low SES more likely to benefit from combination of fluoxetine and CBT or fluoxetine alone relative to CBT alone or placebo (Curry et al,^67^ 2006)
Parent education	1	1 (100)	0	0	0	NA
Single parent household	1	1 (100)	0	0	0	NA
BMI	1	1(100)	0	0	0	NA
Clinical profile						
Depression symptom severity	9	5 (55)	1(11)	1(11)	3 (33)	High severity more likely to benefit from combination fluoxetine and CBT relative to monotherapies or placebo (Curry et al,^67^ 2006; Foster et al,^70^ 2019); lower BDI score more likely to benefit from SNRI relative to SSRI (Asarnow et al,^68^ 2009); if higher severity, more likely to benefit from C-CBT relative to TAU (Merry et al,^48^ 2012)
Anxiety	6	3 (50)	2 (34)	0	1 (17)	If anxiety present, more likely to benefit from IPT-A relative to TAU (Mufson et al,^49^ 2004)
Hopelessness	4	3 (75)	1(25)	0	0	NA
Suicidal ideation	4	3 (75)	0	1 (25)	0	NA
No. of comorbid disorders	4	3 (75)	0	0	1 (25)	If more comorbid conditions, more likely to benefit from CBT with medications relative to medications alone (Asarnow et al,^68^ 2009)
Duration of symptoms	3	3 (100)	0	0	0	NA
Disruptive behavior	3	2 (66)	0	0	1 (33)	If high marital discord, more likely to benefit from combination fluoxetine and CBT or fluoxetine alone relative to CBT alone or placebo; if low marital discord, more likely to benefit from combination fluoxetine and CBT relative to fluoxetine alone, CBT alone, or placebo (Amaya et al,^74^ 2011)
ADHD	3	1 (33)	1 (33)	0	1 (33)	With ADHD, combination of medication and psychotherapy, fluoxetine alone, and CBT alone had similar results, all more likely to benefit relative to placebo; without ADHD, more likely to benefit from combination medication and psychotherapy relative to fluoxetine alone, followed by CBT and placebo (Kratochvil et al,^69^ 2009)
Age of onset of depression	2	2 (100)	0	0	0	NA
Substance use	2	2 (100)	0	0	0	NA
Previous episodes of depression.	1	0	0	0	1 (100)	If prior episodes, more likely to benefit from CBT relative to life skills group (Rohde et al,^103^ 2006)
Sleep disturbance	1	0	0	0	1 (100)	If sleep disturbed, less likely to benefit from fluoxetine relative to placebo (Emslie et al,^85^ 2012)
NSSI present	1	0	1 (100)	0	0	NA
Depressed mood	1	1 (100)	0	0	0	NA
Anhedonia	1	1 (100)	0	0	0	NA
Somatic symptoms	1	1 (100)	0	0	0	NA
Suicidal ideation	1	1 (100)	0	0	0	NA
Observed symptoms	1	1 (100)	0	0	0	NA
Melancholic features	1	1(100)	0	0	0	NA
Family history of depression	1	1 (100)	0	0	0	NA
Dysthymia	1	1 (100)	0	0	0	NA
Psychosocial context						
Impaired family functioning	6	4 (66)	0	0	2 (33)	Better family functioning associated with greater benefit from combination fluoxetine and CBT better relative to fluoxetine alone; better family functioning associated with greater benefit from fluoxetine alone or placebo relative to CBT alone (Feeny et al,^86^ 2009); complex interaction between marital discord, oppositionality, and gender in TADS (Amaya et al,^74^ 2011); impaired family functioning associated with greater benefit from IPT-A relative to TAU (Mufson et al,^49^ 2004)
General functioning	5	4 (80)	0	0	1 (20)	If high impairment in functioning with friends, more likely to benefit from IPT-A relative to TAU (Mufson et al,^49^ 2004)
Trauma and/or childhood adversity	5	2 (40)	0	0	3 (60)	If history of trauma, less likely to benefit from combination of fluoxetine and CBT or fluoxetine alone relative to CBT alone or placebo (Lewis et al,^94^ 2010); more likely to respond more slowly to combination of fluoxetine and CBT or CBT alone (Waldron et al,^110^ 2019); less likely to respond to combination of medication and psychotherapy relative to fluoxetine alone (Foster et al,^70^ 2019); less likely to benefit from CBT with medications relative to medication alone (Asarnow et al,^68^ 2009; Vitiello et al,^109^ 2011; Shamseddeen et al,^105^ 2011); war-affected adolescent girls without a history of abduction more likely to respond to IPT-G relative to WL; boys with no history of abduction less likely to respond to IPT-G relative to WL (Bolton et al,^33^ 2007)
Psychological factors	3	2 (66)	0	0	1 (33)	If high cognitive distortions at baseline, more likely to benefit from combination fluoxetine and CBT relative to fluoxetine alone; and more likely to benefit from fluoxetine alone relative to CBT alone or placebo (Curry et al,^67^ 2006)
Caregiver psychopathology	2	1 (50)	0	0	1 (50)	Less likely to benefit from CBT relative to family therapy or supportive therapy (Brent et al,^29^ 1997)
Medication history	2	1 (50)	1 (50)	0	0	NA
Coping and problem-solving	1	0	0	0	1 (100)	If good coping skills, more likely to respond to CBT relative to life skills group (Rohde et al,^103^ 2006)
Verbal intelligence	1	1 (100)	0	0	0	NA
Treatment expectations	1	0	0	0	1 (100)	If higher treatment expectations, more likely to respond to combination of fluoxetine and CBT relative to fluoxetine alone (Foster et al,^70^ 2019)
Setting	1	1 (100)	0	0	0	NA
Referral source	1	1 (100)	0	0	0	NA
No. of adverse predictors	1	0	0	1 (100)	0	NA
Therapist factors	1	1 (100)	0	0	0	NA

Abbreviations: ADHD, attention-deficit/hyperactivity disorder; BDI, Beck Depression Inventory; BMI, body mass index; CBT, cognitive-behavioral therapy; C-CBT, computerized cognitive behavioral therapy; IPT-A, interpersonal psychotherapy for adolescents; IPT-G, group interpersonal therapy; NA, not applicable; NSSI, nonsuicidal self-injury; RCT, randomized clinical trial; SES, socioeconomic status; SNRI, serotonin norepinephrine reuptake inhibitor; SSRI, selective serotonin reuptake inhibitor; TADS, Treatment for Adolescents with Depression Study; TAU, treatment as usual; WL, waiting list.

### Postbaseline Predictors

Across 16 RCTs, we identified 107 unique variables tested as postbaseline predictors, grouped into 19 variable domains. In these RCTs, variables were tested as postbaseline predictors in 114 instances, and 68 results (60%) were reported as not significant. Postbaseline variable domains reported as significant with respect to depression outcomes in at least 3 RCTs included early response to treatment, sleep changes, and attendance at psychotherapy sessions (Table 4).

**Table 4.** Postbaseline Variables Tested as Predictors of Depression Symptom Severity Outcomes in RCTs of Treatment for Depressive Disorder in Adolescents

Postbaseline variable domain	RCTs testing variable, No.	RCT analyses by result category, No. (%)				RCTs by direction of significant multivariable analyses, No.		
		Not significant	Significant on univariate analysis, dropped on multivariable analysis	Significant on univariate analysis, not challenged in multivariable analysis	Significant on any multivariable analysis	Greater depression symptom severity at end point	Less depression symptom severity at end point	Unclear or mixed results
Clinical profile								
Improvement in sleep or good sleep	4	0	0	1 (25)	3 (75)	0	3	0
Early response	4	0	0	3 (75)	1 (25)	0	1	0
Posttreatment depressive symptoms	2	0	0	0	2 (100)	2	0	0
Posttreatment Beck Hopelessness Scale	2	1 (50)	1 (50)	0	0	0	0	0
Improvement in substance use outcomes	1	0	0	0	1 (100)	0	1	0
Psychosocial context								
Maladaptive psychological factors	3	1 (33)	0	1 (33)	1 (33)	1	0	0
Poor general functioning	2	0	0	1 (50)	1 (50)	1	0	0
Poor family functioning	2	1 (50)	0	0	1 (50)	1	0	0
Treatment factors								
Attendance at psychotherapy	10	5 (50)	0	3 (30)	2 (20)	0	1	1
Medication factors	3	2 (66)	0	0	1 (33)	0	0	1
Treatment-emergent symptoms or adverse events	1	0	0	1(100)	0	0	0	0
CBT homework completion	1	0	0	0	1 (100)	0	1	0
Group facilitator	1	1 (100)	0	0	0	0	0	0
Clinician fidelity to CBT protocol	1	1 (100)	0	0	0	0	0	0
Treatment completion (in all groups)	1	0	0	1 (100)	0	0	0	0
Therapy component exposure	1	0	0	0	1 (100)	0	1	0
Treatment satisfaction	1	0	0	0	1 (100)	0	1	0
Knowledge about depression and its treatment	1	0	0	1 (100)	0	0	0	0
End point during summer break	1	0	0	0	1 (100)	0	1	0

Abbreviations: CBT, cognitive behavioral therapy; RCT, randomized clinical trial.

### Mediators

Only 5 publications^28,61,62,63,64^ across 4 RCTs conducted formal mediation analyses. A total of 16 variables were tested for mediation. In the Treatment for Adolescents with Depression Study (TADS), which compared fluoxetine, CBT, their combination, and placebo among 439 participants, reduction in perfectionism^61^ and increase in active motivation^64^ (in contrast to precontemplative, contemplative, and maintenance stages of change) mediated depression outcomes; however, these relationships were not specific to any treatment group. In a subgroup of Latinx youth studied by Reyes-Portillo and colleagues,^63^ improvements in measures of relationship functioning with peers and family partially mediated the relationship between interpersonal psychotherapy and improvements in depression outcome. Kaufman and colleagues^62^ tested multiple potential mediators of group CBT for adolescents with depression and conduct disorder, including working alliance with the therapist, group cohesion, skill use, dysfunctional attitudes, and automatic thoughts. Only changes in automatic thoughts mediated the depression outcome. Similarly, Smith and colleagues^28^ found that changes in ruminative thinking mediated the benefits of computerized CBT.

### Preliminary Risk of Bias Assessment

eTable 10 in Supplement 2 outlines the results of our preliminary risk of bias assessment. Of the 81 publications, only 12 (15%) reported that at least 1 model evaluating predictors, moderators, and/or mediators was developed a priori; 20 (25%) reported that their models were developed as post hoc tests; and 49 (60%) did not report when the model to be tested was developed. Of the 81 publications, only 10 (12%) reported any adjustment for multiple comparisons; 15 (19%) reported that their models were not adjusted for multiple comparisons; and 56 (69%) did not report on whether adjustments were made for multiple comparisons. Only 2 publications^65,66^ reported both a priori model development and adjustment for multiple comparisons. Each of these articles found that higher baseline symptom severity and higher baseline parent-child conflict were associated with unfavorable depression outcomes in multivariable analyses.

## Discussion

To optimize the application of principles of precision medicine for the management of DD-A, this scoping review is the first to broadly map out the literature with respect to predictors, moderators, and mediators associated with treatment response in RCTs. Most variables reviewed were classified as not significant. Variable domains reported as significant with respect to their association with outcomes in at least 3 RCTs included age, sex/gender, baseline depression severity, early response to treatment, sleep changes, parent-child conflict, overall psychopathology, suicidal ideation, hopelessness, functional impairment, attendance at psychotherapy sessions, and history of trauma. In the 2 studies that indicated a priori model development and adjusted significance levels for multiple comparisons, both baseline symptom severity and baseline parent-child conflict were associated with unfavorable depression outcome. Only 5 publications reported results of mediation analyses, and no mediation findings have been replicated to date between RCTs.

Next steps can include the examination of variables identified in this review in individualized patient data meta-analyses (eg, Zhou et al^118^); this examination may include the use of machine learning strategies in large data sets to clarify their relative importance. If these variables continue to show an important association with outcomes, investing in RCTs designed to specifically examine differential effects of treatment based on the variables is warranted. For example, results from an RCT comparing trauma-focused CBT to depression-focused CBT in adolescents who meet criteria for both DD-A and have a history of trauma could be very helpful in making tailored treatment decisions. To further assess the role of early response or nonresponse to treatment, the use of adaptive trial designs (eg, Gunlicks-Stoessel et al^115^) or the study of measurement-based care (eg, Courtney et al^119^) can also be pursued.

Gaps to be addressed in the literature reviewed here include vulnerability to bias, heterogeneity of findings, and predictors, moderators, and mediators omitted from analyses. Multiple factors can contribute to potential bias. First, analyses of predictors, moderators, and mediators are unlikely to be adequately powered, as sample size calculations are typically made on primary analyses, increasing the probability of type II errors (ie, the risk of not detecting of an important association between independent and dependent variables, when there truly is one).^120^ Moreover, secondary analyses are often at risk of both publication bias and retrospective bias.^121^ The set of predictor, moderators, and mediator analyses in our review are susceptible to these biases in that only 12 of the 81 publications identified reported that their predictor, moderator, and mediator analyses were developed a priori. Next, multiple testing without adjustment of *P* values also renders secondary analyses vulnerable to type I errors (the risk of detecting an association between variables when there is none).^122^ We found that only 10 of the 81 publications made *P* value adjustments for multiple testing. The Bonferroni correction was the only method of adjustment we observed. There is ongoing debate regarding whether the Bonferroni correction is too conservative.^123,124^ Lastly, each variable tested was studied in the context of specific interventions and comparators; results from one trial may or may not be generalizable to adolescents exposed to treatments that were not studied in that specific RCT.

The reported results of the reviewed studies were also quite heterogeneous. Heterogeneity in reported findings was found both between and within RCTs. For example, comorbid anxiety disorder was associated with worse depression outcomes in TADS on multivariable analysis,^67^ but it was not found to be associated with outcomes in the Treatment of Resistant Depression in Adolescents (TORDIA) study.^68^ Within the TADS trial, there was a discrepancy regarding whether a diagnosis of attention-deficit/hyperactivity disorder (ADHD) was associated with differential outcomes between groups across different types of subanalyses^69,70^; 1 article^69^ reported that compared with those without ADHD, participants with ADHD were more likely to have favorable depression outcomes from combined medication and therapy treatment relative to placebo while another article^70^ did not find this association. Within-trial heterogeneity may also be attributed to different measurement instruments being used. For example, Birmaher and colleagues found that poor family functioning at baseline, as measured by the Conflict Behavior Questionnaire,^125^ was associated with unfavorable depression outcome in a large psychotherapy trial,^29^ but another baseline measure of family functioning (the McMaster Family Assessment Device^117^) was not associated with depression outcomes in the same trial.^65^

There are multiple variable domains of interest that have been omitted by the predictor, moderator, and mediator analyses in RCT samples. For example, potential biomarkers of treatment response, like electroencephalogram patterns, functional magnetic resonance imaging findings, inflammatory markers, heart-rate variability, polysomnography findings, genetic markers, and cortisol levels have not been reported in the included RCTs. The role of comorbid borderline personality disorder has also not been reported. The association of treatment implementation factors, like quality and extent of training in psychotherapy, with outcomes has not been examined. The effect of clinician fidelity to the psychotherapy model was only reported in 1 publication^71^ of 11 trials that examined in-person psychotherapy and was reported to be not significant. In psychotherapy trials, the extent to which skill acquisition is associated with depression severity outcomes has also only been evaluated in 1 publication^62^ examining mediators of the effects of group CBT in adolescents with comorbid depression and conduct disorder. These variables all require further exploration.

To improve the quality and clinical usefulness of secondary analyses moving forward, investigators examining predictors, moderators, and mediators should incorporate methods to minimize retrospective and publication bias, increase harmonization of variable domain measurement, and standardize reporting of predictor, moderator, and mediator analyses. To mitigate the effects of retrospective and publication bias, researchers can preregister access to data, research questions, and analysis decision-trees.^126^ A priori documentation of these processes (eg, through Open Science Framework^127^) means that investigators can still have flexibility in their analytic approach, but are less prone to erroneously highlighting interesting results that may lead to research waste. Increasing harmonization of variable domain measurement can be done through the development of a core outcome set, where all prospective trials are collecting a minimum set of common data in a standardized fashion.^128^ This process can facilitate the pooling of data across studies which, in turn, facilitates adequately powered analyses. Such a process is under way.^129^ Investigators would also benefit from the use of widely recognized and standardized guides on how to conduct and report predictor, moderator, and mediator analyses. Recently published guides include The Transparent Reporting of a Multivariable Prediction Model for Individual Prognosis Or Diagnosis (TRIPOD),^130^ the Instrument for Credibility of Effect Modification Analyses (ICEMAN),^22^ and the Guideline for Reporting Mediation Analyses of Randomized Trials and Observational Studies (AGReMA)^131^ checklists.

Authors of recent predictor, moderators, and mediator analysis publications have advised caution with the clinical implications of using results for applying precision medicine to the treatment of DD-A.^70,72^ The high proportion of not significant findings in our review is consistent with this recommendation. The practice of evidence-based medicine integrates the use of the best available evidence, clinician expertise, and patient values.^132^ Clinicians need to acknowledge the very limited evidence currently available to support the practice of precision medicine when treating DD-A and rely more on their own expertise and patient values until the field is further advanced. For most adolescents presenting with DD-A, we advise broadly applying recommendations from high-quality clinical practice guidelines (eg, NICE guidelines) as a starting point.^11,133^ To further personalize care, a shared decision-making model can be used,^134^ in which treatment decisions are made through the active elicitation of patient values, the collaborative discussion of benefits and risks of treatment options in light of these values, and the extent of the evidence to support these options. Measuring response to treatment and changing treatment if there is no response (ie, measurement-based care^135^) can also optimize the personalization of care.

### Limitations

There are a number of limitations to consider in this scoping review. In the absence of an established method for syntheses of predictor, moderator, and mediator analyses, we had to use consensus-based methods to aggregate independent variables into domains and coding strategies. In the absence of a universally recognized authority on what constitutes an adequate statistical model, the models were not assessed for quality and may also be a source of variation in findings. Our coding system also favored highlighting significant findings over not significant ones, as was required to simplify complex findings. Moreover, we relied on the quality of reporting in the publication to code the various analyses. If reporting in a given publication was unclear, it is possible that codes were misclassified with respect to the actual analysis undertaken. Additionally, we categorized results with respect to a *P* value threshold as significant and not significant. There are multiple critiques of using and interpreting *P* values to describe the importance of findings as well as active debate on the use of *P* values altogether.^136,137,138^

## Conclusions

Our scoping review highlights the limited extent to which the literature on predictors, moderators, and mediators can be used to inform further research into precision medicine principles as they apply to the treatment of DD-A. The field would benefit from the use of recognized and established processes and reporting guidelines for predictor, moderator, and mediator analysis publications and related evidence syntheses. In practice, it is important for clinicians to acknowledge uncertainty with respect to matching treatment to patient profiles. Researchers can use information from this review to guide next steps for study design, such as individual patient data meta-analyses, machine learning strategies, and trials of interventions targeting specific variables thought to be associated with unfavorable depression symptom severity outcomes. With high-quality investigations of predictors, moderators, and mediators associated with outcomes through robust research designs, there is immense potential to improve the lives of adolescents with depression.

## Notes

Supplement 1. **eAppendix.** Search Strategy

Supplement 2. **eTable 1.** Randomized Clinical Trials With Predictors, Moderators, and/or Mediators Associated With Outcomes of Treatment of Depression in Adolescents **eTable 2.** Publications Containing Analyses of Predictors, Moderators, and/or Mediators Associated With Depression Outcomes in RCTs for the Treatment of Depression in Adolescents **eTable 3.** Detailed Coding of Variables Tested as Baseline Predictors of Depression Outcomes **eTable 4.** Coding Results Aggregated by Trial and by Variable Domain Tested as Baseline Predictors of Depression Outcome **eTable 5.** Detailed Coding of Variables Tested as Moderators of Depression Outcome **eTable 6.** Coding Results Aggregated by Trial and by Variable Domain Tested as Moderators of Depression Outcome **eTable 7.** Detailed Coding of Variables Tested as Postbaseline Predictors of Depression Outcome **eTable 8.** Coding Results Aggregated by Trial and by Variable Domain Tested as Postbaseline Predictors of Depression Outcomes **eTable 9.** Detailed Coding of Variables Tested as Mediators of Depression Outcomes **eTable 10.** Preliminary Risk of Bias Assessments for Secondary Analyses
