# Supplementary material for: Predictors, Moderators, and Mediators Associated With Treatment Outcome in Randomized Clinical Trials Among Adolescents With Depression: A Scoping Review
Source: JAMA Netw Open. 2022 Feb 1;5(2):e2146331. doi: 10.1001/jamanetworkopen.2021.46331 (PMC8808324; doi:10.1001/jamanetworkopen.2021.46331)
Supplement: Supplement 1. — eAppendix. Search Strategy [file jamanetwopen-e2146331-s001.pdf]

Supplemental Online Content

Courtney DB, Watson P, Krause KR, et al. Predictors, moderators, and mediators associated with treatment outcome in randomized clinical trials among adolescents with depression: a scoping review. *JAMA Netw Open.* 2022;5(2):e2146331. doi:10.1001/jamanetworkopen.2021.46331

eAppendix. Search Strategy

This supplemental material has been provided by the authors to give readers additional information about their work.

© 2022 Courtney DB et al. *JAMA Network Open.*

eAppendix. Search Strategy

Adolescent Depression RCTs (and secondary/meta-analyses of RCTs)

Database: Ovid MEDLINE(R)

Search Strategy:

1 depressive disorder/ or depressive disorder, major/ or depressive disorder, treatment-resistant/ or dysthymic disorder/

2 depress*.ti,kf.

3 dysthymi*.ti,ab,kf.

4 (major adj3 depress*).ti,ab,kf.

5 (diagnos* adj3 depress*).ti,ab,kf.

6 (unipolar adj3 (depress* or mood or disorder*)).ti,ab,kf.

7 or/1-6 [major depression concept]

8 Adolescent/

9 (adolescen* or youth* or teen* or young adult*).ti,kf.

10 or/8-9 [adolescent concept]

11 7 and 10 [depression + adolescents]

12 secondary analys*.af.

13 (posthoc analys* or post-hoc analys* or moderator* or mediator* or predictor*).ti,kf,hw. 14 or/12-13 [secondary analyses type]

15 (meta-analysis or metaanalysis).ti,ab,kf,hw,pt.

16 11 and 14 [depress + adolescents + SA]

17 11 and 15 [depress + adolescents + MA]

18 16 or 17 [depression + adolescents limited to MA + SA]

19 "randomized controlled trial".pt.

20 (random* or placebo* or single blind* or double blind* or triple blind*).ti,ab. 21 (retraction of publication or retracted publication).pt.

22 19 or 20 or 21

23 (random sampl* or random digit* or random effect* or random survey or random regression).ti,ab. not "randomized controlled trial".pt.

24 22 not 23 [BMJ Filter partial]

© 2022 Courtney DB et al. *JAMA Network Open.*

25 11 and 24 [depression + adolescents + BMJ]

26 18 or 25 [teen + depression + SA/MA/BMJ]

27 (animals NOT humans).sh.
28 ((comment or editorial or practice-guideline or review or letter or journal correspondence) NOT "randomized controlled trial").pt.
29 27 or 28

30 26 not 29 [adolescent + depression + SA/MA/BMJ - limits]

© 2022 Courtney DB et al. *JAMA Network Open.*
